# The Potential Role of GLP1-RAs Against Anticancer-Drug Cardiotoxicity: A Scoping Review

**DOI:** 10.3390/jcm14082705

**Published:** 2025-04-15

**Authors:** Filippo Biondi, Rosalinda Madonna

**Affiliations:** Department of Pathology, Cardiology Division, University of Pisa, Via Paradisa, 56124 Pisa, Italy

**Keywords:** cardioncology, antrhacyclines, HER2, GLP1-RA, glucagon-like peptide-1 receptor agonists, semaglutide, exenatide, liraglutide, dulaglutide, tirzepatide, chemotherapy, anti-cancer treatment

## Abstract

**Background:** GLP1 receptor agonists (GLP1-RAs) have become a central component in the treatment of type 2 diabetes mellitus (T2DM) and are gaining prominence in the cardiovascular field. Semaglutide and other GLP1-RA molecules possess cardioprotective properties. Cardiotoxicity, a term used to refer to cardiovascular disease caused by anticancer treatment, is a collection of common and severe conditions. Its pharmacological prevention or mitigation is a clinical unmet need as options are few and limited to some specific clinical settings. GLP1-RAs have a promising pharmacological profile given their activity on a number of pathophysiological targets and signaling pathways including oxidative stress, autophagy, and STAT3 activation. Interestingly, abnormalities in some of the GLP-1-modulated pathways have been linked to cardiotoxicity. This scoping review aims to map the extent and assess the main characteristics of research on the role of GLP1-RAs in the prevention and/or mitigation of anticancer-related cardiotoxicity. **Methods**: The selection process led to the inclusion of thirteen studies chosen from reports retrieved through the search string: (“semaglutide” OR “exenatide” OR “liraglutide” OR “dulaglutide” OR “tirzepatide” OR “GLP1 receptor agonist” OR “GLP1RA” OR “GLP1-RA” OR “GLP1” OR “Glucagon-like Peptide-1 Agonists”) AND (“cardioncology” OR “cardiotoxicity” OR “chemotherapy” OR “anti-cancer treatment” OR “anti-cancer therapy”). The study complied with the PRISMA guidelines on scoping reviews. **Results**: Two studies were clinical and conducted on registries, eight used animal models, two were conducted on cell cultures, and one was conducted on both animal models and cell cultures. Evidence in favor of cardioprotection and a number of putative mechanisms emerged. **Conclusions**: Evidence on GLP1-RAs’ effect on cardiotoxicity is limited in both quantity and quality and suffers from poor study standardization. However, most included studies documented a rigorously defined cardioprotective effect and demonstrated changes in several pathophysiologically relevant targets and pathways, including NF-κB, IL-6, reactive oxygen species, and caspase-3. Further clinical studies are warranted.

## 1. Introduction

Cancer diagnoses have significantly increased in recent decades; however, advancements in treatment options have led to a steady rise in overall survival rates among cancer patients [1]. As survival improves and new therapeutic options and regimens are introduced into clinical practice, the short- and long-term adverse effects of anticancer treatments on the cardiovascular system gain prominence as sources of morbidity and mortality [2]. The mortality from cardiovascular (CV) disease in long-term breast cancer survivors has been shown to exceed that from the initial disease and from cancer recurrence [3,4,5]. Moreover, the incidence of cardiotoxicity in a diversified cohort of cancer patients was nearly 40% in the CARDIOTOX registry [6]. The relevance of this finding is emphasized by the fact that in this registry cardiotoxicity was narrowly defined as cases of new or worsening myocardial damage or cardiac dysfunction. The burden of CV disease as a result of anticancer therapy is even greater in the pediatric population, given its combination of high and growing survival rates, longer life expectancy, and greater susceptibility to its adverse CV effects [7,8].

A wide range of anticancer drugs is involved in cardiotoxicity, a term gathering a variety of cardiac and/or vascular conditions that were recently defined in a consensus statement by the International Cardio-Oncology Society [9]. Regarding cardiotoxic anticancer drug classification, several criteria have been proposed, but the most practical and clinically relevant one is based on the leading clinical and/or instrumental findings that can be associated with each drug class. Anthracyclines and human epidermal growth factor 2 (HER2) inhibitors are primarily linked to heart failure (HF) and cardiac dysfunction; vascular endothelial growth factor (VEGF) inhibitors, mechanistic target of rapamycin (mTOR) inhibitors, and platinum-based therapies to arterial hypertension and venous thrombosis; checkpoint inhibitors to a variety of clinical effects, including vascular, myocardial, and pericardial disease [10]. Moreover, virtually all cardiotoxic drug classes have been linked to arrhythmias, with the notable exception of anthracyclines, antimetabolites, platinum-based therapies, HER2 inhibitors, and vascular endothelial growth factor (VEGF) inhibitors [10].

Regarding management, cardiotoxicity can be mitigated through drug class-specific strategies based on baseline risk assessment, the proper selection of anticancer-drug regimens, and the use of cardioprotective pharmacological therapies. A number of drugs known for their cardioprotective effect have been tested against cardiotoxicity. The latest guidelines from the European Society of Cardiology favor the use of primary pharmacological prevention in patients at high or very high risk of CV toxicity [11]. In particular, dexrazoxane, a chelating agent, should be considered in the prevention of anthracycline cardiotoxicity; angiotensin-converting enzyme inhibitors (ACEis) or angiotensin receptor blockers (ARBs) in anticancer-related heart failure regardless of the drug class involved; statins in all high- or very high-risk patients. Notably, all of these therapeutic measures received a class IIa-B recommendation, except for ACE inhibitors (ACEis), angiotensin receptor blockers (ARBs), and beta-blockers, which have a class IIa-C recommendation if used against anthracyclines and anti-HER2 antibodies. Evidence supporting these recommendations derives from a wide body of preclinical data, randomized controlled trials, and meta-analyses [12,13,14,15]. Although not included in the cited guidelines, several clinical and preclinical studies have suggested that sodium-glucose co-transporter 2 inhibitors (SGLT2is) [16,17] and mineralocorticoid antagonists (MRAs) [12] exert cardioprotection against cardiotoxicity in various conditions and anticancer therapies. Moreover, new evidence suggests that SGLT2i-mediated cardioprotection may also apply to targeted anticancer therapies, such as ponatinib [18,19].

Similarly to SGLT2i, glucagon-like peptide-1 receptor agonists (GLP1-RAs) improve cardiovascular outcomes, especially in metabolic patients, with or without established cardiovascular disease [20,21,22,23]. GLP1-RAs are pleiotropic and have a wide range of target systems and tissues [24]. These effects, which have been shown in both preclinical and clinical studies, may be mediated by a number of mechanisms. In particular, GLP1-RAs stimulate the generation of NO, thereby contributing to the preservation of endothelial function through the activation of the AMPK/Akt/eNOS pathway. They also reduce inflammation by regulating the expression of proinflammatory cytokines, such as IL-6 and TNF-α; negatively modulate apoptosis by decreasing the Bax/Bcl-2 ratio and caspase activation; and sustain the autophagic flux [25]. On a physiological level, GLP1-RAs exert a direct CV activity by reducing heart rate and blood pressure [26,27,28]. Regarding cardioprotection, wide preclinical evidence exists on the ability of GLP1-RAs to preserve ventricular function and reduce the extent of experimentally induced myocardial infarction in animal models ([29,30,31,32]). Of note, the presence of GLP1 receptors was demonstrated by Mclean et al. [33] in mouse cardiomyocytes across all cardiac chambers and in endocardial cells. Moreover, the same study showed that the GLP1-RA-mediated improvement in mouse survival and reduction in infarct extension depended upon the expression of Glp1r Tie2 genes. These findings provide strong biological plausibility to the hypothesis that GLP1-RA exert a direct cardioprotective activity independently of glucose-lowering and other metabolic effects [33]. Turning to clinical research, trials have documented a significant impact of GLP1-RAs on CV clinical endpoints, with a more evident beneficial effect on atherosclerosis and on heart failure with preserved ejection fraction [20]. In particular, a number of trials [34,35,36,37,38] showed a reduction in a composite of CV mortality, non-fatal myocardial infarction, and ischemic stroke in patients with T2DM. Prevalence of HF in these studies was variable, but low [20]. HFpEF has been specifically addressed by four specific large randomized controlled trials that have shown improvements in quality of life [22,23], HF hospitalization [21], and major adverse cardiovascular events [39]. Results were much less encouraging in heart failure with reduced ejection fraction (HFrEF) cohorts, in which liraglutide was associated with a heightened risk of CV rehospitalization [40], ventricular tachycardias, and atrial fibrillation [38,41].

However, cardioprotection in metabolic patients does not necessarily equate to protection against cardiotoxicity. From a mechanistic point of view, GLP1-RAs possess several molecular effects that could theoretically counteract cardiotoxicity from several anticancer treatments. Table 1 shows the correspondence between some putative mechanisms of anthracycline-mediated cardiotoxicity and molecular modifications associated with GLP1-RAs. In particular, as mentioned regarding cardioprotection in general, GLP1-RAs have been shown to reduce oxidative stress, preserve mitochondrial function [42,43], counteract apoptosis [44], and stimulate autophagy [45] and they also seem to exert a favorable effect on endothelial dysfunction [46]. A recent review by Quagliarello et al. [47] offers an in-depth analysis of the pathophysiological background of cardiometabolic outcomes in cancer patients that sets the stage for a possible cardioprotective effect of GLP1-RAs.

This review aims to systematically assess existing preclinical and clinical evidence regarding the potential benefits of GLP1-RAs in anticancer treatments to provide an insight into relevant molecular mechanisms and inform future research in the field of pharmacological prevention of cardiotoxicity.

## 2. Methods

This scoping review was conducted following the Preferred Reporting Items for Systematic Reviews and Meta-Analyses Extension for Scoping Reviews (PRISMA-ScR) guidelines. We set out to include any experimental study testing the effect of any drug of the GLP1-RA class, either in patient cohorts undergoing anticancer treatment or in preclinical models of cardiotoxicity. All anticancer treatments were included, and no restriction was applied regarding measured outcomes or date of publishing. Non-English publications and grey literature were excluded from this review. The search was conducted in PUBMED and EMBASE using the following search string on the 1st of February 2025: (“semaglutide” OR “exenatide” OR “liraglutide” OR “dulaglutide” OR “tirzepatide” OR “GLP1 receptor agonist” OR “GLP1RA” OR “GLP1-RA” OR “GLP1” OR “Glucagon-like Peptide-1 Agonists”) AND (“cardioncology” OR “cardiotoxicity” OR “chemotherapy” OR “anti-cancer therapy”). The two authors independently screened titles and abstracts. Full-text articles were retrieved and assessed for eligibility, with discrepancies resolved through discussion. A standardized data extraction form was used to collect relevant study characteristics and findings, which were predetermined by the two authors and are shown in the header row of Table 2. This review was not registered on PROSPERO, as the platform does not accept scoping reviews. Even though no formal analysis of selection bias or sensitivity analysis was performed, efforts were made to ensure transparency in the selection process by adhering to the PRISMA guidelines.

## 3. Results

**Figure 1:**
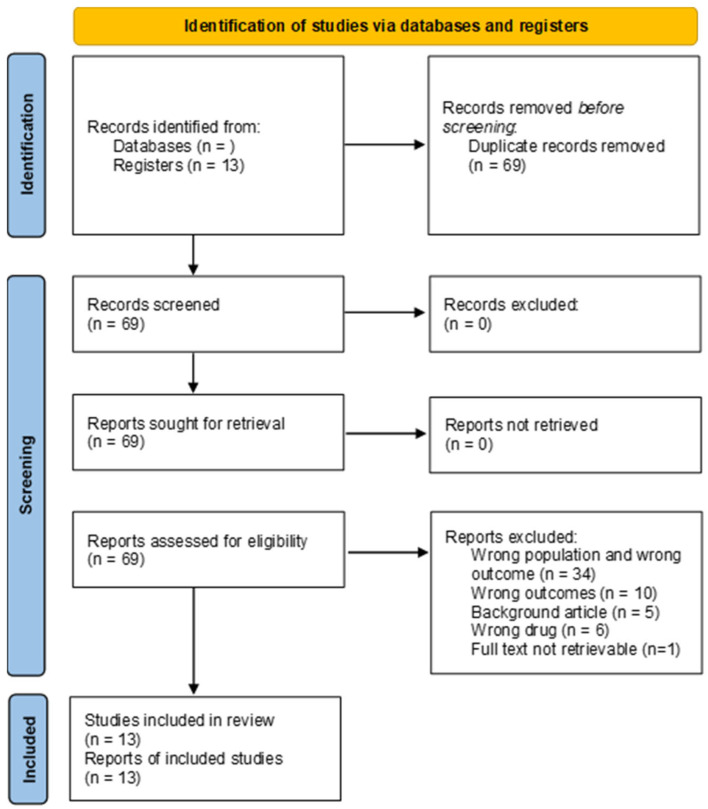
The PRISMA flow diagram [69] showing all phases that led to the selection of pertinent papers.

The study selection process is illustrated in the PRISMA flowchart (Figure 1). The initial search produced sixty-nine results. No records were removed, before or after screening. All reports were effectively retrieved and assessed for eligibility. Fifty-six reports were excluded. Thirty-four of these were excluded because the study model or study population was not appropriate. Ten studies did not measure outcomes pertaining to cardiotoxicity. Five studies were literature reviews, and six studies did not test GLP1-RAs. Thirteen studies were eventually included in the review.

All included studies were conducted between 2017 and 2025. Five studies tested liraglutide, three semaglutide, one exenatide, and one GLP1. Two studies enrolled cohorts of patients treated with more than one GLP1-RA.

**Figure 2:**
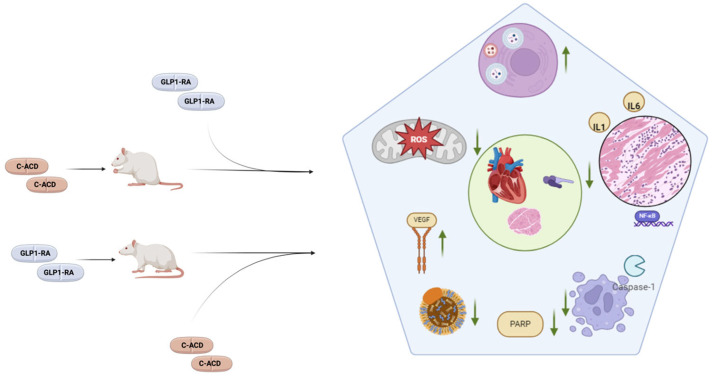
The main mechanisms shown to be positively modulated by GLP1-RAs in animal models treated with cardiotoxic anticancer drugs (C-ACDs). Animals, which were either pre-treated or post-treated with GLP1-RAs, exhibited cardioprotective effects in terms of echocardiographic parameters, cardiac biomarkers, and histological changes (green circle). These were associated to favorable effects on a number of pathways, the main examples of which are represented inside the pentagon. In particular, GLP1-RAs were associated to improved autophagic flux, decreased inflammation, decreases apoptosis, decreased PARP expression, a decline in LDL cholesterol, an increase in VEGF expression, and a decline in oxidative stress.

Regarding study design, two studies were clinical and conducted on registries, eight used animal models, two were conducted on cell cultures, and one on both animal models and cell cultures (Table 2). Regarding animal studies, six studies were conducted on Wistar rats, two on Sprague Dawley rats, and one on mice. Cell cultures consisted of H92C cardiomyoblasts in one study and EA.hy926 endothelial cells in the other. Study endpoints were highly variable. As shown in Table 2, non-clinical studies tracked changes in a diverse range of molecules, pathways, and instrumental findings, while the two clinical studies measured clinical endpoints. Concerning experimental protocols, studies adopted variable numbers and types of control arms. The order and duration of GLP1-RA and anticancer drug administration was variable across studies. Regarding animal studies, the sample size ranged from twenty to sixty. One study did not specify the sample size. All but one study showed a significant positive effect on the investigated endpoints. Eleven studies documented a cardioprotective effect of GLP1-RAs: six through a reduction in markers of myocardial necrosis/overload (cardiac troponins and/or creatine kinase MB and/or brain natriuretic peptide), two through an improvement in echocardiographic parameters, two through histopathological findings, one through an improvement in ECG, and two through the improvement in clinical endpoints. Nine studies offer insights into mechanisms potentially underlying the GLP1-RA cardioprotective effect, as detailed in Table 2. In particular, GLP1-RAs were associated to a reduction in caspase-3 and p53 expression; an increase in the autophagic flux; a decline in tumor necrosis factor alpha (TNF-α), interleukin-1 (IL-1), and interleukin-6 (IL-6); and a rise in superoxide dismutase (SOD) expression. Studies also found a GL1-RA-dependent decline in LDL cholesterol, a reduction in poly ADP-ribose polymerase 1 (PARP), β-galactosidase, and p16INK4A expression. Despite the variability in experimental protocols and GLP1-RA molecules tested, findings were largely consistent. From a pathophysiological point of view, the described changes in investigated pathways substantiate evidence on the cardioprotective role of GLP1-RAs, given their role in anticancer cardiotoxicity [68]. Results are summarized in Figure 2.

## 4. Discussion

The potential role of GLP1-RAs in cardioncology as a preventive strategy for anticancer drug-induced cardiotoxicity has been gaining growing attention. The number of preclinical cellular and animal studies is on the rise, and the first two clinical studies were recently conducted. Moreover, even though most of the investigational effort has been dedicated to anthracycline-mediated cardiac dysfunction, other models of cardiotoxicity have been addressed as well. However, research on the potential role of GLP1-RAs in cardioncology is certainly at a preliminary stage. To put things into perspective, SGLT2is, which were first approved for the therapy of T2DM eight years later than GLP1-RAs, have been tested in a much more extensive manner in cardiotoxicity, and high-quality data have been provided by randomized controlled trials [70] and meta-analyses [71], especially with regard to anthracycline-mediated cardiotoxicity.

The included studies investigated GLP1-RA-mediated cardioprotection through biochemical, instrumental, and histopathological assessments. The large majority of them (eleven out of twelve) showed a statistically significant improvement associated with GLP1-RA treatment. Moreover, the two retrospective observational studies [63,67] showed a positive effect on hard clinical endpoints, including all-cause mortality, with large benefits in terms of hazard ratios. From a general point of view, it is widely known that only a small fraction of preclinical studies identifies associations that ultimately prove to be clinically significant in real-world settings [72]. This has been attributed to biological differences, poor study designs, publication bias, and lack of standardization [73]. Regarding poor standardization, the experimental protocols in the included animal studies were variable in terms of GLP1-RA molecules, anticancer drugs, drug doses, administering schedules, and order of administration (Table 3). It is worth noting that GLP1-RAs were administered as a pre-treatment in four studies, co-administered in three studies, and followed the administration of the anticancer drug in two studies. Such variability in experimental protocols makes it difficult to compare the different studies and draw general conclusions. Moreover, preclinical studies have an intrinsic tendency to overestimate effect sizes because of several reasons, including the idealized conditions in which they are run, publication bias, and small sample size. Included preclinical studies are therefore likely to give an overly optimistic picture of GLP1-RA efficacy in cardiotoxicity and should be evaluated as hypothesis-generating. The same warning applies to cellular studies, which offer an even less realistic representation of drug effects in humans. It should be noted, however, that all the included studies opted for solid, non-surrogate biomarkers of cardiotoxicity, such as troponins and histological changes.

Included studies conducted on animal and in vitro models provide some potentially useful mechanistic insights as well. In particular, they show that GLP1-RAs seem to mitigate anthracycline-induced cardiotoxicity through several mechanisms. These include: an improvement in cell survival (reduced caspase-3 activation); an increase in the autophagic flux, through an increase in AMPK-Akt pathway activity; an amelioration of inflammatory mediators, including TNF-α, IL-1, and IL-6; and a decrease in oxidative stress, as demonstrated by both a decline in ROS and a heightened expression of superoxide dismutase (SOD). Of note, findings were consistent across studies, despite cited differences in GLP1-RA molecules and experimental protocols. HamaSalih et al. [65] were the only researchers to investigate and document a decrease in LDL cholesterol associated with semaglutide, which partially reversed LDL increase caused by doxorubicin. Even though statins are recommended in the primary prevention of cardiotoxicity, little evidence exists concerning the possible effect of anthracyclines on the lipid profile [74], whereas modest LDL reduction is a known effect of GLP1-RAs. Turning to targeted therapies, the only included study investigating gefitinib-mediated cardiotoxicity found a reduction in poly ADP-ribose polymerase 1 (PARP) expression, associated with decreased inflammation (reduced NF-κB) and oxidative stress (improved SOD expression). PARP has been linked to cardiotoxicity [43] and PARP inhibitors have been effectively tested for cardioprotection [44,75]. The ability of GLP1-RAs to downregulate PARP was already shown in a study by Li et al. [76] and would be worth further research. Regarding methotrexate, VEGF inhibition is a known mechanism of cardiotoxicity [10], but it has also been suggested to mediate methotrexate’s pharmacodynamic action [77], coherently with the notion that angiogenesis is a fundamental aspect of cancer biology. GLP1-RAs were shown to upregulate VEGF, which represents a plausible mechanism of cardioprotection but could also theoretically reduce the therapeutic efficacy of methotrexate. This same possibility applies to the effect of semaglutide and GLP-1 on cisplatin and fluorouracil cardiotoxicity. In particular, semaglutide was found to counteract apoptosis in cisplatin-treated rats through a decrease in caspase-3 and p-53 expression. Caspase-3 is a well-known mediator of cisplatin-induced apoptosis [78]. Similarly, fluorouracil-induced senescence, defined as an increase in the expression of beta-galactosidase, an increase in p16INK4A, and a reduction in cell proliferation, was attenuated by GLP-1. Cell senescence has been shown to be associated to cardiovascular disease [79] and could represent a valuable therapeutic target in its treatment and prevention. However, its role is less clear in cancer. Senescence is indeed thought to be a natural barrier to tumorigenesis [80] and has been suggested to act as a marker and possible mediator of anticancer drug efficacy [81]. The counteraction of the beneficial effects of anticancer drugs on apoptosis, VEGF expression, and senescence suggests a potential interference of GLP1-RAs with specific anticancer therapies. However, this remains a purely mechanistic speculation, as no study has specifically examined the impact of GLP1-RAs on cancer-related outcomes or delved into the possibility that GLP1-RAs may interfere with anticancer drugs.

Regarding clinical evidence of GLP1-RAs’ cardioprotective effect, the review only included two retrospective, observational, registry-based studies. Both were conducted on the same source, i.e., the TriNetX research network registry. Both studies found a very large effect size on risk ratios for MACEs and all-cause mortality, which is rarely seen in RCTs in the CV space. In fact, retrospective observational studies are often plagued by bias and confounding from unknown factors [82].

Future research needs to rely on clinical studies so as to investigate whether the encouraging results provided by animal experiments correspond to a clinically relevant effect in the real world. New retrospective observational studies may gather valuable information in the short term by leveraging data provided by registries and databases. The prevalence of T2DM in cancer is as high as 8–18% [83] and a large part of these patients is treated with GLP1-RAs. However, considering the intrinsic limitations of observational studies due to confounding, definitive causal inference requires randomization. It would be therefore essential to run randomized controlled trials which could initially concentrate on patients with cancer and concomitant metabolic and/or atherosclerotic disease. As said, GLP1-RAs have shown the most promising cardiovascular effects in this setting [84]. Trials will also have to pay adequate attention to safety outcomes, concerning both CV and cancer-related outcomes. This is particularly important in the light of the known safety signals associated to the use of GLP1-RAs in HFrEF and also due to the mechanistic insight that some favorable molecular effects of anticancer drugs may be counteracted by GLP1-RAs [61,62,85]. The clinical impact of GLP1-RA-mediated weight loss on cancer prognosis will also have to be evaluated. The prognostic impact of cachexia on cancer patients is very severe [86] and GLP1-RAs have been found to determine more muscle loss than other means of weight loss [87].

## 5. Conclusions

Evidence on GLP1-RAs’ effect on anticancer drug cardiotoxicity is still in the preliminary phase and is limited to cellular, animal, and retrospective registry studies. The included papers, however, shed light on a number of molecular mechanisms that might be at play, while providing plausible evidence of cardioprotection against anticancer drugs. Regarding the translational potential of existing evidence, no clinical recommendation can be made on the basis of the included studies, but further research is warranted, especially considering GLP1-RAs’ wide use and pleiotropic profile. Future efforts should aim at conducting observational and randomized controlled trials to back the existing exploratory studies with rigorous proof of clinical benefit. Due attention will have to be paid to safety outcomes as well, considering that GLP1-RA-mediated weight loss may worsen cachexia and the potential interference of GLP1-RAs with anticancer treatment efficacy.

## Figures and Tables

**Table 1:** Table showing the correspondence between some mechanisms of anthracycline cardiotoxicity and GLP1-RA pharmacological profile.

Mechanism of Anthracycline Cardiotoxicity	Supporting References	GLP-1 RA Activity	Supporting References
Oxidative Stress, ROS Formation, and Mitochondrial Function	Carrasco L. et al. [48]	Reduces ROS and oxidative stress	Clara Luna-Marco et al. [49]
Endothelial Dysfunction and Microvascular Injury	Sawyer et al. [50]	Protects endothelial cells	Rossella Menghini et al. [51]
Inflammation and Apoptotic Pathways Activation	Hutchins et al. [52]	Reduces inflammatory markers	Giulia Bendotti et al. [53]
Autophagy Dysregulation	Russo et al. [54]	Modulates autophagy	Sarah Costantino et al. [55]

**Table 2:** The table header shows the variables for which data were sought in the scoping review. Each row presents the references and main characteristics of each included study.

Paper Title	First Author and Reference	Year of Publication	Study Type	Endpoints	Evidence of Cardioprotection	Mechanicistic Insights/Molecular Findings
Enhanced-autophagy by exenatide mitigates doxorubicin-induced cardiotoxicity	Kyung Hye Lee, et al. [56]	2017	Cell culture (H9C2 cardiomyoblast) + animal study (Sprague Dawley rats).	Cell viability, ROS generation, autophagic flux, echocardiographic parameters.	Recovery of ejection fraction and fractional shortening.	Increase in autophagic flux; Reduced caspase-3 activation; AMPK activation; Reduced ROS.
Liraglutide ameliorates cardiotoxicity induced by doxorubicin in rats through the Akt/GSK-3β signaling pathway	Noha A. T. Abbas, Soad L. Kabil [57]	2017	Animal study (Wistar rats).	CK-MB, troponin I, SOD, MDA, TNF-α, IL-6, GSK-3β, AMPK, p-Akt, Bcl-2 expression.	Decrease in troponin I, CK-MB.	Increase in SOD, AMPK, p-Akt activity. Decrease in MDA, IL-6, TNF-α, GSK-3β, TGF-β1, and caspase-3. Increased Bcl-2 expression. Reduction in inflammation and necrosis. Reduction in TGF-β.
5-Fluorouracil Causes Endothelial Cell Senescence: Potential Protective Role of GLP-1	Paola Altieri, et al. [58]	2017	Cell culture (EA.hy926 endothelial cells).	Cell senescence, eNOS, SIRT-1, PKA, and PI3K pathway activation.	//	Decreased senescence. Reduced eNOS and SIRT-1.
Therapeutic Effects of Liraglutide, Oxytocin, and Granulocyte Colony-Stimulating Factor in Doxorubicin-Induced Cardiomyopathy Model	Emin Taskiran, et al. [59]	2019	Animal Study (Sprague Dawley rats).	ECG, MDA, TNF-α, troponin T, pro-BNP, caspase-3.	Decrease in troponin T and pro-BNP.	Caspase-3 immunosuppression. Reduction in plasmaTNF-α.
Liraglutide attenuates gefitinib-induced cardiotoxicity and promotes cardioprotection through the regulation of MAPK/NF-κB signaling pathways	Abdullah F. AlAsmari, et al. [60]	2020	Animal study (Wistar albino rats).	ECG, biochemical markers, histology.	Decrease in troponin, CK-MB, NT-pro-BNP.	Reduction in NF-κB. Reduced PARP expression. Increase in SOD expression. KNK and p38 phosphorylation.
Assessment of the cardioprotective effect of liraglutide on methotrexate-induced cardiac dysfunction through suppression of inflammation and enhancement of angiogenesis in rats	R.H. Mahmoud, et al. [61]	2021	Animal study (Wistar albino rats).	ECG, biochemical markers, histology.	Improved ECG change; normalized histopathological changes.	Increased VEGF expression. Decreased IL-6 and IL-1β.
The cardioprotective effect of human glucagon-like peptide-1 receptor agonist (semaglutide) on cisplatin-induced cardiotoxicity in rats: Targeting mitochondrial functions, dynamics, biogenesis, and redox status pathways	Marwa Mohamed Atef, et al. [62]	2023	Animal study (Wistar rats).	Mitochondrial function, dynamics, biogenesis, redox status, apoptosis.	Decrease in CK-MB and LDH.	Increase in SOD expression. Attenuation of PINK1 and parkin mRNA overexpression. Elevation of PGC-1. Reduced p53 expression. Reduction in caspase-3 expression.
Glucagon-like Peptide-1 Agonists Reduce Cardiovascular Events in Cancer Patients on Immune Checkpoint Inhibitors	Cho han Chiang, et al. [63]	2023	Retrospective, clinical study.	Major Adverse Cardiovascular Events (MACEs), mortality.	Reductions in myocardial infarction or need for coronary revascularization, heart failure, and all-cause mortality.	//
Liraglutide Pretreatment Does Not Improve Acute Doxorubicin-Induced Cardiotoxicity in Rats	Carolina R. Tonon, et al. [64]	2024	Animal study (Wistar rats).	Echocardiogram, isolated heart functional study.	No improvement at echocardiogram.	//
Effects of Semaglutide in Doxorubicin-Induced Cardiac Toxicity in Wistar Albino Rats	Raz Muhammed HamaSalih, et al. [65]	2024	Animal study (Wistar rats).	Serum biochemical markers (troponin, CPK, LDL, etc.); histopathological analysis.	Decrease in CPK and troponin; improved vascular congestion and inflammation.	Decrease in LDL cholesterol.
Semaglutide attenuates doxorubicin-induced cardiotoxicity by ameliorating BNIP3-Mediated mitochondrial dysfunction	Xiaoping Li, et al. [66]	2024	Animal study (mice).	Cardiac function, mitochondrial function.	Decrease in CK-MB, BNP. Improvement at echocardiogram.	BNIP3 reduction through activation of PI3K/AKT pathway.
The Role of GLP-1 Receptor Agonists in Managing Cancer Therapy-Related Cardiac Dysfunction (MEdRxiv Preprint)	Aravinthan Vignarajah, et al. [67]	2025	Retrospective clinical study (TriNetX research network registry).	Composite of heart failure exacerbation, death, and admission to the hospital or emergency department; mortality rate; heart failure; all-cause hospitalization.	Reduction in composite outcome; improved survival decrease in mortality rate, heart failure, and all-cause hospitalisation.	//

**Table 3:** Table giving an overview of the variability in the design and protocol of the included studies.

Study	Authors and Reference	Dose and Administration	Order and Time Delay
Enhanced-autophagy by exenatide mitigates doxorubicin-induced cardiotoxicity	Kyung Hye Lee, et al. [56]	Exenatide: 10 μg/kg, subcutaneous injection, every 2 days for six doses. Doxorubicin: 20 mg/kg cumulative dose, intraperitoneal injection.	Exenatide first, followed by doxorubicin after 1 h; once every 2 days, six total times, euthanasia at 28th day.
The cardioprotective effect of human glucagon-like peptide-1 receptor agonist (semaglutide) on cisplatin-induced cardiotoxicity in rats: Targeting mitochondrial functions, dynamics, biogenesis, and redox status pathways	Marwa Mohamed Atef, et al. [62]	Cisplatin: 2 mg/kg/day, intraperitoneal injection for 1 week. Semaglutide: 12 μg/kg, subcutaneous injection once daily for 8 weeks after cisplatin treatment.	Cisplatin first for 1 week, followed by semaglutide daily for the next 8 weeks.
Assessment of the cardioprotective effect of liraglutide on methotrexate-induced cardiac dysfunction through suppression of inflammation and enhancement of angiogenesis in rats	R.H. Mahmoud, et al. [61]	Liraglutide: 300 μg/kg/day, subcutaneously for 10 days. Methotrexate: 20 mg/kg, intraperitoneally as a single dose on day 10.	Methotrexate first, on day 10, followed by liraglutide daily for 10 days.
Liraglutide Pretreatment Does Not Improve Acute Doxorubicin-Induced Cardiotoxicity in Rats	Carolina R. Tonon, et al. [64]	Liraglutide: 0.6 mg/kg/day, subcutaneously for 14 days. Doxorubicin: 20 mg/kg, intraperitoneally on day 12.	Liraglutide daily for 14 days, and doxorubicin on day 12 (only for D and DL. groups).
Effects of Semaglutide in Doxorubicin-Induced Cardiac Toxicity in Wistar Albino Rats	Raz Muhammed HamaSalih, et al. [65]	Semaglutide (low dose): 0.06 mg/kg or 0.12 mg/kg/day or 0.24 mg/kg/day, subcutaneously for 7 days. Doxorubicin (DOX): 12 mg/kg, intraperitoneally on day 7.	Semaglutide daily for 7 days, followed by doxorubicin on day 7.
Semaglutide attenuates doxorubicin-induced cardiotoxicity by ameliorating BNIP3-Mediated mitochondrial dysfunction	Xiaoping Li, et al. [66]	Doxorubicin: 5 mg/kg, intraperitoneally once a week for 4 weeks. Semaglutide: 12 μg/kg, subcutaneously daily for 6 weeks.	Semaglutide daily for 6 weeks, with doxorubicin given in four intermediate weeks.
Therapeutic Effects of Liraglutide, Oxytocin, and Granulocyte Colony-Stimulating Factor in Doxorubicin-Induced Cardiomyopathy Model	Emin Taskiran, et al. [59]	Doxorubicin: 2.5 mg/kg/day, intraperitoneally every other day for six doses (total 15 mg/kg). Liraglutide: 1.8 mg/kg/day, intraperitoneally for 15 days. Oxytocin: 160 μg/kg/day, intraperitoneally for 15 days. Filgrastim: 100 μg/kg/day, intraperitoneally for 15 days.	Semaglutide and doxycycline co-administered for 15 days.
Liraglutide ameliorates cardiotoxicity induced by doxorubicin in rats through the Akt/GSK-3β signaling pathway	Noha A. T. Abbas, Soad L. Kabil [57]	Doxorubicin: 1.25 mg/kg, intraperitoneally, four times per week for 4 weeks. Liraglutide: 100 μg/kg, intraperitoneally daily for the following 4 weeks.	Doxorubicin administered 4×/week for 4 weeks, followed by liraglutide daily for 4 weeks.
Liraglutide attenuates gefitinib-induced cardiotoxicity and promotes cardioprotection through the regulation of MAPK/NF-κB signaling pathways	Abdullah F. AlAsmari, et al. [60]	Liraglutide: 200 mg/kg, intraperitoneally, once daily. Gefitinib: 30 mg/kg, orally, once daily.	Liraglutide pre-administered daily for 7 days, followed by co-administration of liraglutide and gefitinib daily for 21 days.

## Abbreviations

GLP1-RA: Glucagon-like Peptide 1 Receptor Agonist; T2DM: Type 2 Diabetes Mellitus; HFpEF: Heart Failure with Preserved Ejection Fraction; SGLT2i: Sodium-Glucose Cotransporter 2 inhibitor; ACEi: Angiotensin-Converting Enzyme inhibitor; ARB: Angiotensin Receptor Blocker; SOD: Superoxide Dismutase; MACE: Major Adverse Cardiovascular Event; PRISMA-ScR: Preferred Reporting Items for Systematic Reviews and Meta-Analyses Extension for Scoping Reviews; LDL: Low-Density Lipoprotein; NF-κB: Nuclear Factor kappa-light-chain-enhancer of activated B cells; VEGF: Vascular Endothelial Growth Factor; PARP: Poly (ADP-Ribose) Polymerase; RCT: Randomized Controlled Trial, HER2: human epidermal growth factor 2.
